# Speech of Dr. Davis at Long Island

**Published:** 1865-07

**Authors:** 


Mayor Lincoln was frequently interrupted with most emphatic applause and enthusiastic cheering. After partial silence had been restored, the Mayor called upon Dr. Davis, President of the Association, who was loudly cheered upon taking the stand—or chair.

He said that he was exceedingly happy to meet the gentleman under circumstances so pleasing to each individual around him. The assembly, he said, almost overwhelmed him with recollections of the past. The historic old city of Boston, which has sometimes been called the cradle of liberty, everything around us, not only the old shaft on Bunker Hill; but the old church, the old graveyard, even the by-ways and crooked ways [laughter] moved him with pleasing recollections of the past. He was happy as he looked around him, as he stood with his feet, as it were, in the eastern extreme of our glorious Union, to see many who would recollect with him a similar excursion to this, west of the Mississippi, and the kind attentions which the Association received there. At this time it should not be forgotten that away in the distant South, even in South Carolina, this Association has assembled and has received the same kind attentions. He would not dare to allude to this, even as a reminiscence, did he not know that the valor of our soldiers brought the South to our fold once again. [Applause.] He said that when his mind travelled over scenes and the pleasing reminiscences of the past years of the life of this Association, which has met in every section of our country, his heart was too full, and his tongue could not express his feelings. We may meet, continued the speaker, for good, to rack our brains in devising better medicines to alleviate the sufferings of mankind, but after all it is the social element in our conventions that makes them so attractive to us. In conclusion, Dr. Davis cordially thanked the City Council, in the name of the National Medical Society, for the generous attention extended to them.

Dr. Woodward, of the Surgeon-General's office, at Washington, was then introduced. He spoke concerning the medical profession in the army, of their perilous position, and their devotion to their duties, saying that had it not been for the aid which our armies have received from the profession they would have been able to do but little in this war.

Dr. Storer, the newly elected President of the Association, was then called for. He was introduced to the gentlemen by His Honor the Mayor in rather a peculiar manner, which caused much laughter. Dr. Storer, in speaking of his election as President of the Association, said that he had hoped that some other gentleman would have been chosen to represent the National Medical Association than himself. He said that he had not the presumption to suppose that he possessed qualifications that were not possessed by the great majority of the members of the Association, but he received the honor as a compliment to Massachusetts. Dr. Storer spoke of the social influence of these conventions. Dr. Worthington Hooker, of Connecticut, made a short speech, speaking in a happy manner of his State, Connecticut, and of the generous manner in which the Association had been treated by the City of Boston. Rev. Dr. Lothrop was the next speaker. In speaking of the medical profession, he said that in his opinion it stands at the top of the professions, and demands the highest gratitude from the infinite and incalculable service which it renders to mankind. Dr. Sayre, of New York, in a short speech, acknowledged the hospitality with which the convention has been received in Boston. Short speeches were also made by Dr. Doherty, of Connecticut, Dr. J. P. Ordway, of Boston, Dr. Curry, of Westchester, N.Y., and others.

About half-past eight o'clock, the party again embarked on the return "voyage." As the steamers left the wharf, rockets and other fireworks were discharged from their decks, and the bands performed favorite airs. The sail home by moonlight was exceedingly pleasant, and the time was occupied in listening to the fine music of the bands, and to singing, conversing, &c. As the steamers passed the pilot boat No. 7, that vessel burned brilliant blue-lights, which were noticed with cheering. On landing at Commercial Wharf, the passengers of the Rose Standish formed in procession and marched to the Tremont House to the music of Gilmore's band, where they separated.

The Committee of Arrangements of the City Council deserve much credit for the admirable manner in which the arrangements for the excursion were carried out. Everything that could be thought of for the comfort and enjoyment of the company was provided.

PROCEEDINGS OF THE FOURTH DAY.

The Association was called to order at 9 o'clock, by the President. Reports from sections were received and appropriately referred, and it was voted that such bills as might be presented therein should be paid by the Treasurer of the Association.

After the transaction of some unimportant business, Drs. Leidy, F. G. Smith, and H. Hartshorn were appointed a committee on the paper of Dr. Corse, on "the microscope."

Dr. J. P. Ordway of Boston, presented a protest against the action of the Association in adopting the resolutions expelling Dr. Montrose A. Pallen. After the words of the resolution the document reads as follows:—

*Whereas*, the association has refused by vote to reconsider its action in this behalf, now, therefore,

We, the undersigned, in attendance, while we execrate the crime with which Dr. Pallen stands charged, and would, if his guilt were established, most promptly and heartily vote for his expulsion, do most respectfully protest against the action of this association, as above recited, on the general principle that all sense of right and justice, as well as the established rules of this association, demand that no man shall be condemned and punished until his guilt is established. And we desire that this protest shall be placed upon the record in the minutes of the proceedings of this annual meeting.

Signed by Drs. James F. Hibberd, Henry I. Bowditch, George B. Twitchell, and about thirty other gentleman.

The ground taken in the protest was ably defended by Dr. Ordway, and after some discussion, on motion of Dr. Hunt, of New Jersey, a special committee of three, consisting of Drs. Hunt, Bibben, of New York, and Baxter, U.S.A., were appointed to prepare an answer to the paper.

A letter from Dr. W. H. Mussey, of Cincinnati, was read by the President, which severely censured the "course of Surgeon-General Barnes, in consulting with a homœopath in the case of Secretary Seward and son, and allowing a quack to prescribe medically whilst he was attending surgically," characterizing it as an "offence of no mean proportions; the high position of the parties making the demoralizing effect the greater." The facts alleged in the letter were declared to be without foundation by Dr. Woodward, of Washington.

